# Allergen Immunotherapy management during vaccinations: An international survey

**DOI:** 10.1016/j.waojou.2021.100601

**Published:** 2021-11-08

**Authors:** Simonetta Masieri, Claus Bachert, Pedro Ojeda, Chang-Keun Kim, Carlo Cavaliere, Giorgio Ciprandi, the Study Group on AIT & Vaccinations

**Affiliations:** a Department of Oral and Maxillofacial Sciences, Sapienza University of Rome, Rome, Italy; b Upper Airways Research Laboratory and Department of Oto-Rhino-Laryngology, Ghent University, Ghent, Belgium; c Division of ENT Diseases, CLINTEC, Karolinska Institute, University of Stockholm, Stockholm, Sweden; d First Affiliated Hospital, Sun Yat-sen University, International Airway Research Center, Guangzhou, China; e Asthma and Allergy Clinic Drs. Ojeda, Madrid, Spain; f Asthma and Allergy Center, Inje University-Sanggye Paik Hospital, Seoul, South Korea; g Department of Sense Organs, Sapienza University of Rome, Rome, Italy; h Allergy Clinic, Department of Outpatients, Casa di Cura Villa Montallegro, Genoa, Italy

**Keywords:** Allergen immunotherapy, Vaccinations, Safety, Survey, Experts

## Abstract

Vaccination against viral and bacterial pathogens represents a challenging issue in allergic subjects, mainly concerning patients undergoing allergen immunotherapy (AIT). For this reason, an international survey has been performed involving a panel of experts who responded to a series of questions, also concerning the COVID-19 impact on allergen immunotherapy and vaccinations.

The results showed that co-administration of vaccines and AIT requires caution, mainly during the pandemic era. Moreover, the choice of AIT product should be oriented considering also the safety profile.

The relationship between allergy and infectious diseases has always been intriguing. In this regard, vaccination against viral and bacterial pathogens represents a challenging issue in allergic subjects, mainly concerning patients undergoing allergen immunotherapy. Surprisingly, this topic is still neglected as very few studies have addressed it. In this regard, Ulrich and colleagues analysed the data of 875 patients receiving subcutaneous immunotherapy (SCIT) and/or vaccination.^1^ The simultaneous vaccination and SCIT did not induce systemic reactions. Consequently, the authors concluded that despite the guidelines recommend an intermission of at least 1 week between SCIT and vaccination, a shorter or no interval could be considered.

The COVID-19 pandemic has overwhelmingly recycled this issue. Two main aspects have been stressed: AIT management during the COVID-19 era and allergic reaction to COVID-19 vaccines. Namely, a warning has been established for severe allergic reactions to both mRNA and non-mRNA COVID-19 vaccines.^2^ The production of specific IgE toward some excipients of these vaccines has been recognized. In particular, the most common allergens were polyethylene glycol 2000 and tromethamine, contained in mRNA vaccines; whereas polysorbate 80 was contained in DNA vaccine.^3^

Moreover, concerns occurred also for thromboembolism from vector vaccines based on modified, replication-deficient adenoviruses. The pathogenic mechanisms are multifactorial and might include also non-IgE mediated pathways, including antibodies against platelet factor 4 (PF4), direct interaction between adenoviral vector and platelets, cross-reactivity of antibodies against adenovirus and PF4, antibody cross-reactivity between SARS-CoV-2 spike protein and PF4, interaction between spike protein and platelets, expression by platelets of spike proteins, or expression of adenoviral proteins by platelets.^4^ In addition, myocarditis is another non-IgE mediated severe adverse reaction to COVID-19 vaccines. The pathogenetic mechanisms are not yet determined, but it is thought that cardiac injury might occur due to inflammatory system hyperactivation and release of inflammatory mediators.^5^ Moreover, non-mRNA COVID-19 vaccines caused severe allergic reactions, including anaphylactic reactions, in a very limited number of subjects.^6^

Therefore, the European and American scientific societies provided detailed recommendations concerning AIT management.^6^^,^^7^ In particular, emotional distress frequently accompanies AIT patients during the pandemic.^8^ Anxiety is widespread and affects adherence to the AIT schedule. In this regard, under the framework of the European Academy of Allergy and Clinical Immunology (EAACI), a group of experts conducted a web-based retrospective survey on practical and safety aspects of AIT during the COVID-19 era.^9^ The outcomes showed that AIT starting was postponed in 60% of the participants, and maintenance AIT was continued by 75% (89% for sublingual immunotherapy, SLIT). Notably, no tolerability concerns arose from the survey. Therefore, a general AIT underutilization occurred.

On the other hand, children treated with AIT and in maintenance dose experienced less severe symptoms during influenza than children in building AIT.^10^ This outcome underlined AIT positive effects on respiratory infections. Moreover, the live attenuated influenza vaccine was safe and well-tolerated by children with moderate to severe asthma.^11^

However, the relationship between vaccines and AIT has not been adequately addressed even now. Based on this background, an international survey explored the practical aspects of AIT and vaccination management during the COVID-19 pandemic. The survey consisted of a series of questions collected in a web-questionnaire administered to a panel of 56 internationally skillful experts in AIT management. The criteria for recruiting the experts were a large experience on the AIT management, based on thousands of patients, and scientific expertise, based on published papers. The panel included 36 allergists, 9 paediatricians, 9 otolaryngologists, and 2 pulmonologists. The participants were from Italy (21), Germany (10), Portugal (8), Spain (6), Albania (4), South Korea (3), Austria (1), Belgium (1), Mexico (1), and United Kingdom (1).

The questionnaire included 14 practical questions as reported in Table 1. All participants completed the questionnaire.

**Table 1:** Questionnaire concerning the management of allergen immunotherapy (AIT) during vaccinations

Question	Answers
Do you have experience of your patients being vaccinated for infectious diseases during AIT?	**Yes 95%**
How many patients did follow?	**Mean 206 (range 2 >3000)**
Do you believe, in your experience, that there could be a negative interference between vaccinations and AIT?	**No 95%**
Do you change the AIT schedules during concomitant vaccinations?	**No 58%**
Do you stop the AIT before vaccination and restart it later?	**No 77%**
If your patients haven't stopped AIT, even accidentally, have there been any issues?	**No 100%**
Did you observe adverse reactions to AIT during vaccinations?	**No 98%**
If yes, what adverse reactions to AIT did you observe?	**Usually local reactions, rarely systemic reactions, very rarely anaphylaxis (only one doctor)**
Did you observe adverse reactions to vaccinations during AIT?	**No 87%**
If yes, what adverse reactions to vaccinations did you observe?	**Mild local reaction alone**
When AIT patients have to be vaccinated, do you behave the same way with SLIT or SCIT?	**No 57%**
Do you consider vaccination for COVID-19 similar to that for other infectious diseases?	**Yes 70%**
Do you provide patients who have to be vaccinated for COVID-19 the same indications that you would give them for the other vaccinations?	**Yes 84%**
During this COVID-19 pandemic and under vaccination for this virus, do you prescribe products considering their safety profiles (no documented severe reactions, no anaphylaxis)?	**Yes 75%**

The large majority (95%) had an experience of about 200 patients being vaccinated for infectious diseases during AIT. Only 5% thought that vaccines negatively affect AIT. Consistently, more than half (58%) did not change the AIT schedule around vaccination, and 77% did not stop AIT before vaccination and never occurred adverse reactions to vaccine without stopping AIT. Equally, almost all (98%) never observed adverse reactions to AIT during vaccinations. Only one participant reported common local reactions but rare systemic ones. Consistently, 87% did not observe adverse reactions to vaccines during AIT. If adverse reactions occurred, they were mild local reactions alone. About half of the participants (57%) believed that SLIT and SCIT require a different approach.

As concerns COVID-19 vaccines, a large quote (70%) of experts considered these vaccinations similar to other vaccinations, consistently the patients' recommendations were usually (84%) the same as far as for other vaccines. The last question concerned the safety profile of AIT. Most participants (75%) pay attention to the safety profile as they prefer to prescribe products with documented safety (absence of severe reported reactions, including anaphylaxis).

Therefore, the current international survey underlined the clinical relevance of the relationship between AIT and vaccinations, mainly during the COVID-19 era. In particular, managing allergic patients who have to be vaccinated is very common in clinical practice. A negative impact of vaccines on AIT is a popular belief, but, surprisingly, many participants did not modify the AIT schedule closely to vaccination. Probably, this behaviour could derive from the almost complete absence of adverse events after vaccinations without stopping AIT.

Another interesting topic concerned the difference between SCIT and SLIT: in fact, about half of the experts behaved differently, believing safer SLIT than SCIT.

Also, nearly three-quarters of the participants considered the COVID-19 vaccines similar to other vaccines. This attitude might depend on the vast impact reserved to this topic by the scientific community and media. However, the suggested indications for patients are often the same as for other vaccinations. Finally, the COVID-19 pandemic induced to think about the safety of AIT products. Most experts preferred to prescribe AIT products, also considering the safety profile. In this regard, a recent study analysed the pharmacovigilance data of an allergoid and reported a meagre rate of adverse reactions.^12^ Consistently, another recent paper underlined that the choice of SLIT compounds should minimize the risk of severe adverse events, mainly concerning anaphylaxis.^13^

This web-based survey had some methodological biases, including the limited number of involved countries, and the selection of participants. These factors affect the outcomes as it did not consider the point of view of relevant countries, such as United States, and continents such as Oceania and Africa. However, the current collection of expert opinions provided interesting information about practical AIT management during the COVID-19 pandemic.

In conclusion, an international panel of experts thought vaccinations during AIT deserve adequate attention even though an actual interference seems to be relatively rare. Moreover, the expert panel retained that AIT products' safety profile represents an important issue in clinical practice.

## Abbreviations

AIT, allergen immunotherapy; COVID-19, coronavirus disease 2019; EAACI, European Academy of Allergy and Clinical Immunology; SCIT, subcutaneous immunotherapy; SLIT, sublingual immunotherapy.

## Funding

No funding.

## Availability of data

Data are in a repository file available on request.

## Ethics approval

Respondents to the survey consented to participation.

## Authors’ contribution

MS designed the study, BC, OP, C-KK, CC discussed the manuscript, GC wrote the paper. All authors approved the manuscript.

## Authors’ consent to publication

All authors consented to the publication of this work.

## Declaration of competing interest

Nothing to declare.

## References

[1] Ullrich D., Ullrich K., Mussler S., Thum-Oltmer S. (2015). Vaccination during concurrent subcutaneous immunotherapy: safety of simultaneous application. Eur Ann Allergy Clin Immunol.

[2] Banerji A., Wickner P.G., Saff R. (2021). mRNA vaccines to prevent COVID-19 disease and reported allergic reactions: current evidence and suggested approach. J Allergy Clin Immunol Pract.

[3] Cabanillas B., Novak N. (2021). Allergy to COVID-19 vaccines. A current update. Allergol Int.

[4] Rzymski P., Perek B., Flisiak R. (2021). Thrombotic thrombocytopenia after COVID-19 vaccination: in search of the underlying mechanism. Vaccines.

[5] Levin D., Shimon G., Fadlon-Derai M. (2021). Myocarditis following COVID-19 vaccination - a case series. Vaccine.

[6] Klimek L., Jutel M., Akdis C. (2020). Handling of allergen immunotherapy in the COVID-19 pandemic: an ARIA-EAACI statement. Allergy.

[7] Shaker M.S., Oppenheimer J., Grayson M. (2020). COVID-19: pandemic contingency planning for the allergy and immunology clinic. J Allergy Clin Immunol Pract.

[8] Celik I.K., Metbulut A.P., Uneri O.S., Dinc G.S., Misirlioglu E.D. (2021). Effect of patient and parental anxiety on adherence to subcutaneous allergen immunotherapy during COVID-19 pandemic. Ann Allergy Asthma Immunol.

[9] Pfaar O., Agache I., Bonini M. (2021). COVID-19 pandemic and allergen immunotherapy – an EAACI Survey. Allergy.

[10] Li Y., Wang D., Zhi L. (2021). Respiratory tract infections in children with allergic asthma on allergen immunotherapy during influenza season. Sci Rep.

[11] Turner P.J., Fleming L., Seglani S. (2020). Safety of live attenuated influenza vaccine (LAIV) in children with moderate to severe asthma. J Allergy Clin Immunol.

[12] Compalati E., Incorvaia C., Urbano S., Strada P., Frati F. (2020). The safety of carbamylated monomeric allergoids for sublingual immunotherapy. Data from pharmacovigilance study. Immunotherapy.

[13] Moesges R., Passali D., Di Gioacchino M. (2021). Worldwide surveys on anaphylaxis to sublingual immunotherapy with house dust mite tablets are urgently needed. Clin Transl Allergy.

